# New applications of cardiopulmonary exercise testing parameters characterize age-related changes in exercise performance during adolescence

**DOI:** 10.3389/fcvm.2026.1751702

**Published:** 2026-06-02

**Authors:** Katerina Kourpas, Takeshi Tsuda

**Affiliations:** 1 Nemours Cardiac Center, Nemours Children’s Health Delaware, Wilmington, DE, United States; 2 Department of Pediatrics, Sidney Kimmel Medical College at Thomas Jefferson University, Philadelphia, PA, United States

**Keywords:** exercise performance, growth, oxygen uptake kinetics, puberty, sex differences, submaximal exercise

## Abstract

**Background:**

Age-related changes in exercise performance during adolescence are complex because of progressive physical and functional changes, in which multiple factors are involved.

**Methods:**

Peak and submaximal cardiopulmonary exercise testing (CPET) parameters were studied in healthy children and adolescents.

**Results:**

Total 303 subjects were divided into six groups by ages and sex: ≤11 years old (yo) (28/22 for males/females), 12–15 yo (61/98), and ≥16 yo (52/42). Rest and peak heart rate (HR) were comparable in all groups. Absolute peak values of oxygen consumption ($\dot{V}$O2), work rate (WR), and oxygen pulse (OP) were higher in older age groups in both sexes, proportionally to the larger body size. When weight-indexed, these peak values were comparable in all male groups but were significantly lower in older female groups ≥12 yo than in ≤11 yo group. Submaximal slope parameters, oxygen uptake efficiency slope (OUES) and Δ$\dot{V}$O2/ΔHR (oxygen pulse slope), demonstrated the same age-dependent trend as peak $\dot{V}$O2 in both sexes. The effects of weight on p$\dot{V}$O2 were progressively enhanced with age only in males, suggesting higher muscle mass and enhanced oxidative metabolism in older males. Exercise endurance beyond anaerobic threshold was augmented in older ages, more prominent in males, indicated by the relationship between ventilatory anaerobic threshold and p$\dot{V}$O2. Age-dependent augmentation in ventilatory oxygenation efficiency was only noted in males.

**Conclusion:**

Exercise performance during adolescence involves multiple factors reflecting somatic growth, functional maturation, and puberty-related body compositions. Significant sex differences were noted in older adolescents.

## 1 Introduction

Cardiopulmonary exercise testing (CPET) is performed to assess overall functional reserve of the cardiovascular system as well as the pulmonary, musculoskeletal, and autonomic nervous systems that participate in oxygen delivery to working muscles (1). Peak CPET values, especially peak oxygen consumption (p$\dot{V}$O2), are highly predictive of daily functionality, quality of life, and prognosis of certain heart diseases (2). Through CPET, functional cardiac reserve will be quantitatively measured, providing more advanced information regarding cardiovascular wellness, especially in patients with heart disease (3). However, contemporary pediatric CPET analysis is subject to multiple confounding factors including pubertal stages, sex, body composition, and physical conditioning (4).

Submaximal CPET parameters represent a dynamic response to early and intermediate phases of exercise (5). Because an achievable peak exercise level can be influenced by multiple factors including patient motivation, physical conditioning, and potential risks induced by peak exercise, i.e., ischemia or arrhythmia, alternative CPET-derived indices have been developed to estimate functional reserve at exercise intensity below maximal aerobic capacity (6). Submaximal CPET parameters are independent of reaching a peak exercise level or peak exercise effort but are often used to predict peak exercise level (6–8).

Age-related growth and maturation bring various changes in body composition and functional capacities, which collectively affect overall exercise performance in adolescents compared with pre-pubertal children. These changes are expressed differently in males and females (9, 10). Males tend to demonstrate higher height, increased muscle mass and strength, increased stroke volume (heart size), increased lung volume, and higher hemoglobin levels than females (9, 11). Females tend to present with increased body fat during the course of puberty in addition to less prominent somatic growth than males (12). Furthermore, age-related growth and functional maturation occur in multiple organ systems independent of sex-related maturation (13, 14). Although the effects of pubertal changes on exercise performance in growing adolescents have been well studied (9, 10, 15), current standard CPET analysis may not sufficiently provide the explanation of underlying mechanisms of exercise performance. This is, in part, due to the fact that body weight is routinely used for scaling in clinical practice to normalize CPET values (16).

The aim of this study is to characterize the exercise performance in growing adolescents and to delineate the underlying physiology of age- and sex-related differences in exercise performance by incorporating peak and submaximal parameters in standard clinical CPET.

## 2 Patients and methods

A retrospective, single-centered, cross-sectional chart review of ordinary children and adolescents who underwent CPET was conducted. All procedures were performed in accordance with hospital guidelines and regulations.

### 2.1 Patients

Healthy individuals without heart disease or other specific medical problems were recruited from the Exercise Laboratory of the Nemours Cardiac Center, Nemours Children's Health Delaware Database from 2005 to 2023. These patients underwent CPET for evaluation of symptoms with or without exercise (e.g., chest pain, palpitation, dizziness, and dyspnea) or minor electrocardiographic abnormalities (e.g., premature ventricular contractions, first degree atrioventricular block, or borderline QT prolongation) and were discharged from further cardiology follow up with clearance of any pathological conditions. Anthropometric measurements including height (cm), weight (kg), and body mass index (BMI: kg/m^2^) were obtained at the time of CPET. Patients with a BMI > 30 kg/m^2^ were excluded from the study. The enrolled patients were divided into three groups by age in both males and females: ≤11 years old (yo), 12–15 yo, and ≥16 yo.

### 2.2 CPET

The CPET was performed via an electronically braked cycle ergometer following the RAMP (Raise, Activate, Mobilize, Potentiate/Performance) protocol with continuous incremental work rate (WR) increase (15–25 watts/min, usually set at approximately 0.3 Watts/kg). Heart rate (HR) and oxygen saturation (%) were continuously monitored by a 12 lead-electrocardiogram and percutaneous pulse oximeter, respectively, throughout the study. Blood pressure (BP) was recorded every 2–3 min during exercise and recovery phases. During CPET, minute ventilation ($\dot{V}$E), $\dot{V}$O2, carbon dioxide production ($\dot{V}$CO2), and respiratory exchange ratio (RER: $\dot{V}$CO2/$\dot{V}$O2) were measured. Oxygen pulse (OP) was calculated by $\dot{V}$O2/HR. These parameters were presented as both absolute and weight-indexed values except $\dot{V}$E which was indexed by height. Anaerobic threshold (AT) was determined by the V-slope method (17). All the above parameters were obtained at AT and peak exercise (VO2 at AT was presented as VAT). Peak exercise effort was determined by achievement of either pRER ≥ 1.05 or peak HR ≥ 90% of estimated peak HR for age (=220 − age).

The submaximal slope parameters were obtained by plotting two values simultaneously to assess slope values up to AT, independent of peak exercise effort.

- Oxygen uptake efficiency slope (OUES)—Ventilatory efficiency for oxygenation. Slope of log_10_[$\dot{V}$E (L/min)] and $\dot{V}O2$ (mL/min) (18)
- Δ$\dot{V}$O2/ΔHR or Δ[$\dot{V}$O2/kg]/ΔHR—Oxygen pulse slope (stroke volume)
- ΔHR/ΔWR or ΔHR/Δ[WR/kg]—HR dependency (HR response to work)
- Δ$\dot{V}$O2/ΔWR—Aerobic work efficiency
- ΔVE/Δ$\dot{V}$CO2—Ventilatory equivalent of CO2

**Figure 1:**
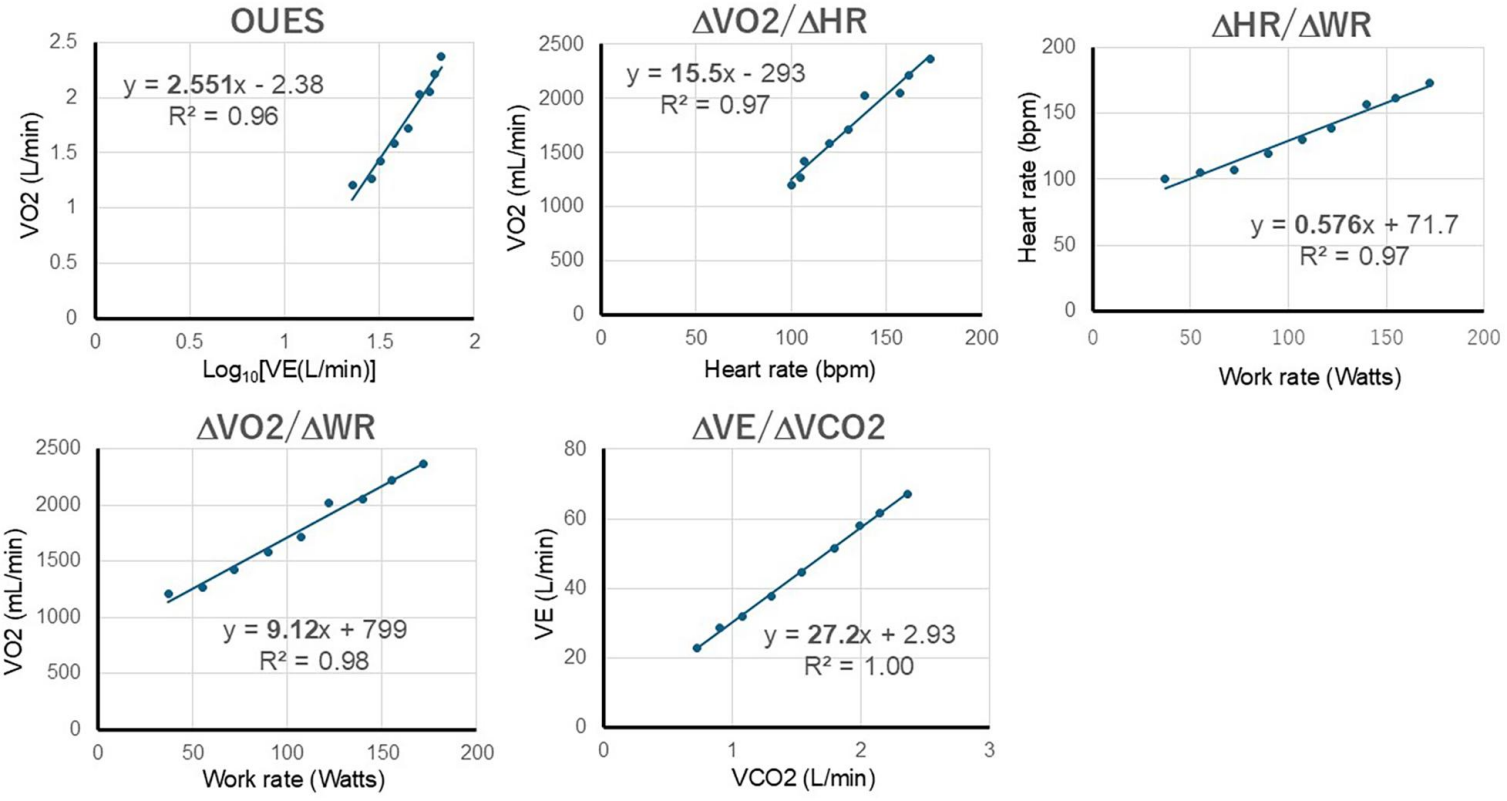
Submaximal parameters of cardiopulmonary exercise testing (CPET). The slopes were obtained during a stable early to intermediate exercise phase up to anaerobic threshold (AT). The patient was a 16 yo male adolescent (weight 80.3 kg).

An example of these submaximal slope parameters is shown in Figure 1.

Selected two CPET parameters were simultaneously plotted in *x*- and *y*-axis (“Two-dimensional analysis”). Scatter plots were obtained from a dataset of one group, from which a regression line was drawn that represents the overall trend of the group. With comparison with regression lines, we aimed to characterize how three different age groups revealed exercise performance. Linear correlations between weight (kg) and p$\dot{V}$O2 (L/min), pWR (watts) and p$\dot{V}$O2 (L/min), VAT (L/min) and p$\dot{V}$O2 (L/min), and p$\dot{V}$E (L/min) and p$\dot{V}$O2 (L/min) were examined, which indicate efficiency of oxygen utilization in skeletal muscle, aerobic work efficiency, exercise endurance beyond AT, and ventilatory efficiency for oxygenation, respectively (19–22).

### 2.3 Statistics

Distribution of patients' demographics and CPET parameters were compared between the three subgroups in each sex and between age-matched males and females. Continuous variables were presented as mean ± standard deviation (SD). Quantitative measure of variability by SD supports a normal variation of the data. Two-sample *t*-test was used to compare the means of continuous variables. One-way analysis of variance (ANOVA) was used to compare the variables between the three groups. Analysis of covariance (ANCOVA) was used to compare the linear regression lines representing groups. Statistical analysis was performed by GraphPad Prizm 6 (GraphPad, San Diego, CA). A *p* value less than 0.05 was determined as statistically significant.

## 3 Results

### 3.1 Demographic profile

The CPET of 303 subjects ranging in age from 7 to 18 years (141 males and 162 females) was studied (Table 1). Enrolled subjects were divided into six groups based on their sex and age: male ≤ 11 yo (*n* = 28), female ≤ 11 yo (*n* = 22), male 12–15 yo (*n* = 61), female 12–15 yo (*n* = 98), male ≥ 16 yo (*n* = 52), and female ≥ 16 yo (*n* = 42). Weight and height show progressive increase with age in both sexes. Males ≥ 16 yo exhibit significantly higher BMI than the younger male groups whereas females ≤ 11 yo show significantly lower BMI than the older female groups.

**Table 1:** Cardiopulmonary exercise testing (CPET) in children and adolescents.

Subgroups	Male			Female		
	≤11 yo	12–15 yo	≥16 yo	≤11 yo	12–15 yo	≥16 yo
Age (years)	10.2 ± 1.0	13.6 ± 1.1	16.5 ± 0.6	9.6 ± 1.2	14.3 ± 0.8	16.6 ± 0.6
Number	24	34	27	20	41	19
Weight (kg)	42.2 ± 12.1	55.6 ± 14.1^†^	69.1 ± 12.5^†^^,^^§^	37.5 ± 7.8	56.1 ± 9.2^†^	60.5 ± 7.8^†^
Height (cm)	145 ± 10	164 ± 11^†^	175 ± 8^†^^,^^§^	144 ± 11	161 ± 6^†^	165 ± 6^†^
BMI [kg·(m^2^) ^−1^]	19.9 ± 4.5	20.3 ± 3.7	22.5 ± 3.2	17.8 ± 1.8	21.5 ± 2.9^†^	22.2 ± 2.8^†^
Rest HR (bpm)	75 ± 13	74 ± 12	69 ± 16	82 ± 12	83 ± 16	76 ± 11
Peak HR (bpm)	182 ± 12	187 ± 14	186 ± 10	187 ± 12	187 ± 10	181 ± 9
%ΔHR	245 ± 37	256 ± 43	274 ± 53	236 ± 36	249 ± 48	237 ± 39
Rest SBP (mmHg)	101 ± 9	111 ± 13	117 ± 31	99 ± 9	104 ± 10	109 ± 15
Peak SBP (mmHg)	140 ± 17	161 ± 24^†^	225 ± 48^†^^,^^§^	136 ± 13	148 ± 16^†^	156 ± 15^†^
%ΔSBP	137 ± 9	144 ± 16	158 ± 16^†^^,^^§^	138 ± 10	143 ± 17	142 ± 15
Peak $\dot{V}$O2 (L·min^−1^)	1.67 ± 0.33	2.42 ± 0.56^†^	3.11 ± 0.82^†^^,^^§^	1.46 ± 0.20	1.89 ± 0.39^†^	1.90 ± 0.35^†^
Peak $\dot{V}$O2/kg (mL·kg^−1^·min^−1^)	40.8 ± 7.6	44.8 ± 7.6	44.5 ± 6.8	39.8 ± 6.3	34.1 ± 5.9^†^	31.5 ± 5.5^†^
Peak WR (Watts)	109 ± 22	170 ± 43^†^	225 ± 48^†^^,^^§^	87.9 ± 16.8	137 ± 27^†^	138 ± 25^†^
Peak WR/kg (Watts·kg^−1^)	2.69 ± 0.68	3.11 ± 0.58^†^	3.26 ± 0.45^†^	2.40 ± 0.46	2.46 ± 0.1	2.51 ± 0.41
peak OP (mL)	9.2 ± 1.6	13.2 ± 3.0^†^	17.0 ± 3.2^†^	7.9 ± 1.1	10.2 ± 2.0^†^	10.6 ± 1.8^†^
Peak OP/kg (mL·kg^−1^)	0.23 ± 0.04	0.24 ± 0.04	0.25 ± 0.03	0.21 ± 0.03	0.18 ± 0.03^†^	0.18 ± 0.03^†^
Peak $\dot{V}$E (L·min^−1^)	62 ± 13	89 ± 22^†^	117 ± 3.1^†^^,^^§^	54.5 ± 11.3	70.2 ± 16.9^†^	70.0 ± 14.5^†^
Peak $\dot{V}$E/height (L·min^−1^m^−1^)	42.3 ± 7.8	53.7 ± 13.2^†^	66.7 ± 17.1^†^^,^^§^	37.7 ± 8.0	43.5 ± 9.6^†^	42.4 ± 9.2^†^
Peak RER	1.13 ± 0.07	1.18 ± 0.08	1.20 ± 0.09^†^	1.13 ± 0.06	1.18 ± 0.06^†^	1.21 ± 0.09^†^
VAT (L·min^−1^)	1.67 ± 0.33	1.58 ± 0.43^†^	1.86 ± 0.46^†^^,^^§^	1.08 ± 0.16	1.31 ± 0.29^†^	1.22 ± 0.36
VAT/kg (mL·kg^−1^·min^−1^)	30.0 ± 6.6	29.7 ± 7.7	26.8 ± 4.8	29.5 ± 4.6	23.7 ± 5.5^†^	20.2 ± 5.8^†^
OUES*	1,815 ± 397	2,519 ± 618^†^	3,079 ± 659^†^^,^^§^	1,660 ± 244	2,103 ± 424^†^	2,196 ± 643^†^
OUES/kg	44.5 ± 8.4	47.0 ± 10.9	44.4 ± 7.1	45.3 ± 7.5	38.1 ± 7.5^†^	36.4 ± 9.8^†^
Δ$\dot{V}$O2/ΔHR (mL)	15.6 ± 2.6	19.7 ± 4.8^†^	23.8 ± 4.8^†^^,^^§^	12.8 ± 0.6	15.1 ± 4.1	15.6 ± 3.8^†^
Δ[$\dot{V}$O2/kg]/ΔHR (mL/kg)	0.36 ± 0.09	0.36 ± 0.07	0.35 ± 0.07	0.35 ± 0.06	0.29 ± 0.07^†^	0.26 ± 0.06^†^
ΔHR/ΔWR [(Watts˒min)^−1^]	0.87 ± 0.17	0.56 ± 0.17^†^	0.45 ± 0.09^†^^,^^§^	1.02 ± 0.24	0.67 ± 0.17^†^	0.62 ± 0.10^†^
ΔHR/Δ[WR/kg] [kg· (Watts˒min)^−1^]	34.9 ± 8.9	29.2 ± 5.1^†^	30.7 ± 5.9	37.2 ± 6.3	36.5 ± 8.1	37.6 ± 7.3
Δ$\dot{V}$O2/ΔWR [mL·(Watts˒min) ^−1^]	12.7 ± 1.8	10.9 ± 1.5^†^	10.7 ± 1.2^†^	12.7 ± 1.3	10.5 ± 1.7^†^	9.8 ± 2.0^†^
Δ$\dot{V}$E/Δ$\dot{V}$CO2	27.4 ± 3.5	24.9 ± 2.9^†^	23.5 ± 2.4^†^	27.3 ± 1.8	25.5 ± 2.8^†^	24.0 ± 3.2^†^
p$\dot{V}$O2/VAT	1.37 ± 0.26	1.58 ± 0.32^†^	1.71 ± 0.30^†^	1.35 ± 0.12	1.48 ± 0.24^†^	1.62 ± 0.27^†^

BMI, body mass index, HR, heart rate, OUES, oxygen uptake efficiency slope, SBP, systolic blood pressure, $\dot{V}$O2, oxygen consumption, WR, work rate, OP, oxygen pulse, $\dot{V}E$, minute ventilation, RER, respiratory exchange ratio, VAT, ventilatory anaerobic threshold, $\dot{V}$CO2, carbon dioxide production.

*Unit of OUES is mL/min/log_10_[L/min].

† *p* < 0.05 compared with ≤11 yo group.

§ *p* < 0.05 compared with 12–15 yo group.

### 3.2 CPET parameters

The CPET parameters are presented in the latter part of Table 1. Both rest and peak HR are comparable in all three age groups in both sexes, but %HR increase (%ΔHR) is significantly higher only in ≥16 yo male group than the younger male groups; females show comparable %ΔHR in all groups. There is a progressive increase in peak systolic BP (SBP) with age in both sexes with no significant difference seen between 12 and 15 yo and ≥16 yo groups in females. Systolic blood pressure increase (%ΔSBP) is significantly elevated only in ≥16 yo males compared with the younger male groups whereas there is no significant difference by age groups in femaless.

Absolute peak CPET parameters, including p$\dot{V}$O2, pWR, pOP, and p$\dot{V}$E, increase progressively with age in male groups, in parallel with an increase in weight and height with age. In females, these parameters are significantly higher in ≥12 yo groups than in ≤11 yo group with no significant difference between 12–15 yo and ≥ 16 yo groups. The weight-indexed values, p$\dot{V}$O2/kg and pOP/kg, are comparable among all male groups but are significantly lower in ≥12 yo female groups than in ≤11 yo group with no significant difference between 12–15 yo and ≥16 yo. Peak WR/kg is significantly higher in ≥12 yo male groups than in ≤11 yo male group but is comparable among all age groups in females. Peak RER is higher in older age groups of both sexes. Peak $\dot{V}$E/height increases significantly with ages in parallel with weight, height, and peak $\dot{V}O2$.

There is no age-related difference in VAT/kg, OUES/kg or Δ[$\dot{V}$O2/kg]/ΔHR in male groups, whereas ≥12 yo female groups show significantly lower OUES/kg and Δ[$\dot{V}$O2/kg]ΔHR than in ≤11 yo female group. The age-related trends of OUES/kg and Δ[$\dot{V}$O2/kg]ΔHR are in parallel with those of p$\dot{V}$O2/kg and pOP/kg in both sexes. In males, ΔHR/ΔWR shows progressive decline with age in accordance with progressive age-related increase of Δ$\dot{V}$O2/ΔHR; a similar but less prominent trend is seen in females. Δ$\dot{V}$O2/ΔWR, aerobic work efficiency or oxygen cost, is significantly lower in ≥12 yo groups than in ≤11 yo groups in both sexes.

### 3.3 Two-dimensional CPET analysis

#### 3.3.1 Weight (kg) and p$\dot{V}$O2 (L/min)

**Figure 2:**
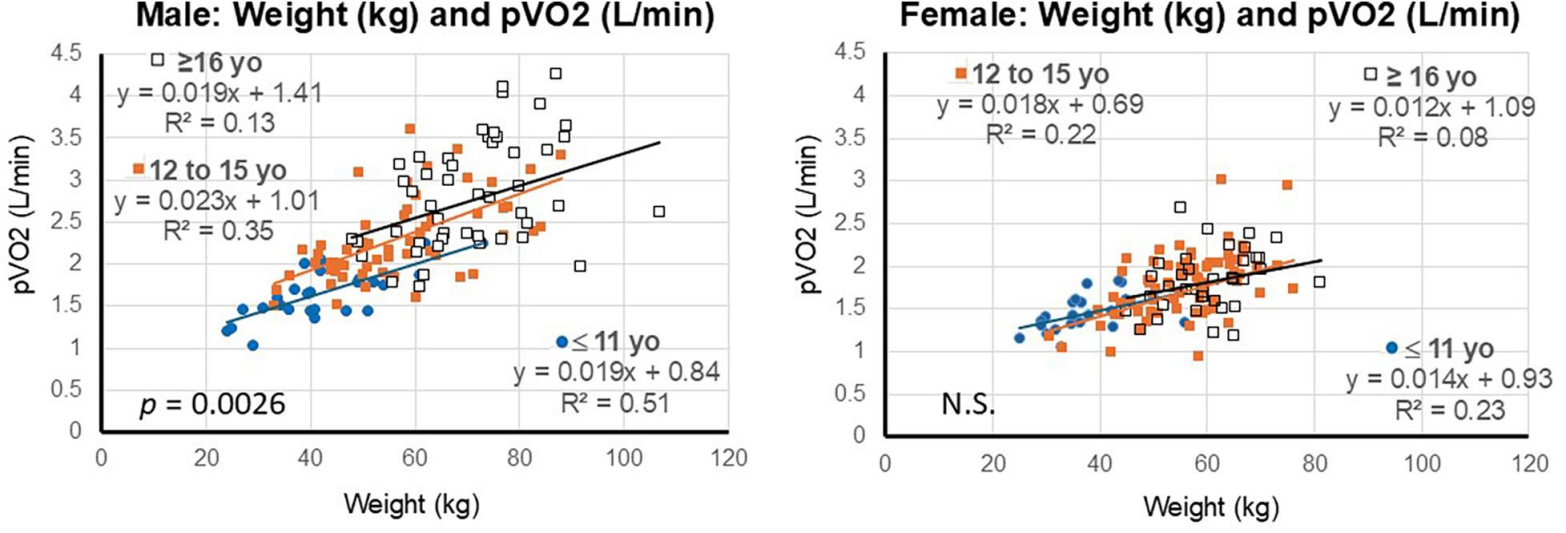
Scattered graphs with regression lines between weight (kg; *x*-axis) and peak oxygen consumption (p$\dot{V}$O2) (L/min; *y*-axis) in males and females with different age groups, indicating differences in efficiency of peak exercise performance by body weight. Male groups show upward deviation of the regression line with increase in age whereas this difference is not seen in female groups. Blue closed circle: ≤11 yo, orange closed square: 12–15 yo, and open square: ≥16 yo.

Figure 2 presents correlations between weight and p$\dot{V}$O2 in different age groups. In male groups, there are good positive linear relationships where a regression line deviates upward as the age advances. This trend suggests more efficient peripheral oxygen uptake in older males, most likely due to higher muscle contents per weight. In female groups, in contrast, the regression lines nearly overlap one another in all 3 age groups. The correlation between weight and p$\dot{V}$O2 is poorer in ≥16 yo groups in both sexes (*R*^2^ = 0.13 and 0.08 in males and females, respectively), likely reflecting more heterogeneous body habitus and physical conditioning in older age groups.

#### 3.3.2 pWR Watts and p$\dot{V}$O2 (L/min)

**Figure 3:**
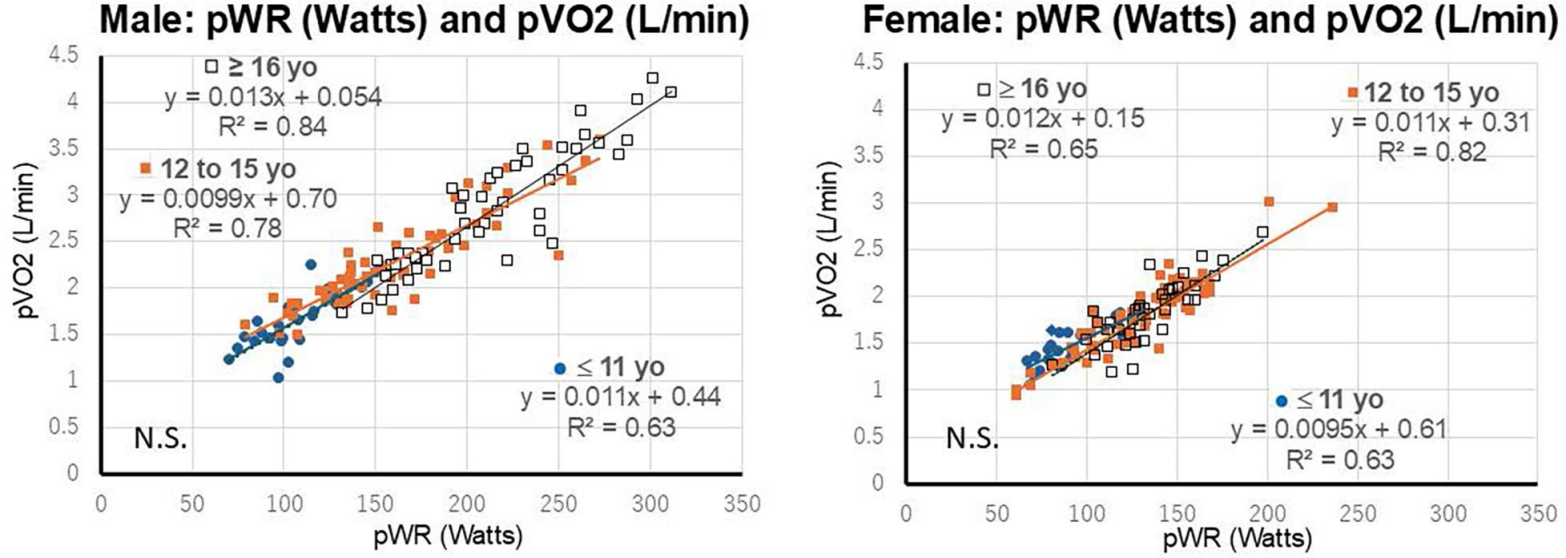
Scattered graphs with regression lines between peak work rate (pWR) (watts) and peak oxygen consumption (p$\dot{V}$O2) (L/min). The regression lines almost overlap one another in both males and females. There is a progressive rightward and upward shift of scatter plot of older age groups in males whereas scatter plot distribution is comparable between 12–15 yo and ≥16 yo in females, concordant with Table 1. Blue closed circle: ≤11 yo, orange closed square: 12–15 yo, and open square: ≥16 yo.

Figure 3 shows excellent positive correlations between pWR (Watts) and p$\dot{V}$O2 (L/min) in all six groups. All three regression lines nearly overlap each other in both sexes, but the ranges of scatter plot distribution reveal a progressive rightward and upward shift with increasing age and body size. Slope values are nearly identical in all 6 groups (male from 0.0099 to 0.013 and females from 0.0095 to 0.012). These trends confirm a consistent correlation between WR and $\dot{V}$O2 in all different age groups independent of sex, age, body habitus, and probably the level of physical conditioning.

#### 3.3.3 VAT and p$\dot{V}$O2

**Figure 4:**
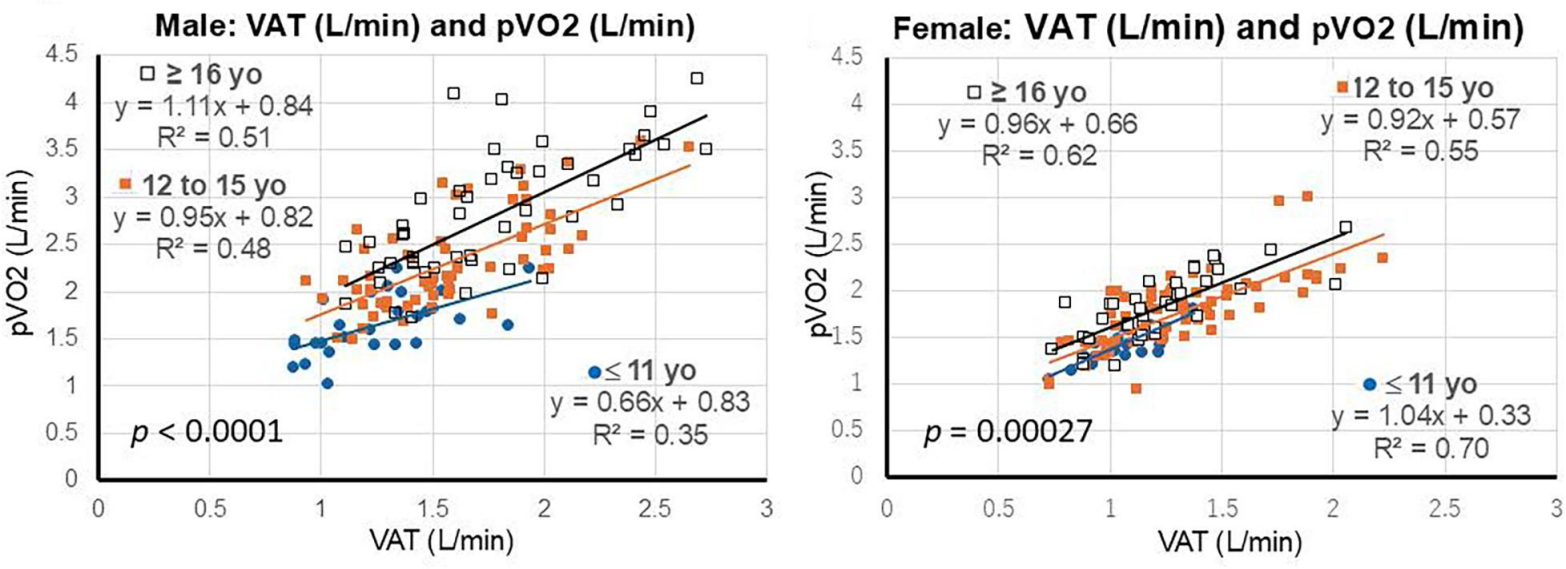
Scattered graphs with regression lines between ventilatory anaerobic threshold (VAT) (L/min; *x*-axis) and peak oxygen consumption (p$\dot{V}$O2) (L/min; *y*-axis) showing the exercise endurance beyond anaerobic threshold (AT). When comparing the absolute values, there is a marked upward shift of the regression lines with increasing age in males whereas this difference was modest in females. Blue closed circle: ≤11 yo, orange closed square: 12–15 yo, and open square: ≥16 yo.

The relationship between VAT and p$\dot{V}$O2 shows a good positive correlation in all age groups, both males and females (Figure 4). In males, there is a progressive upward deviation of the regression lines with increasing age, whereas this age-related upward deviation is relatively modest in females. In males, higher p$\dot{V}$O2 is expected at the same VAT in older ages, suggesting enhanced exercise tolerance under an anaerobic condition that supports longer exercise duration beyond AT. These results are concordant with p$\dot{V}$O2/VAT in Table 1.

#### 3.3.4 pVE and p$\dot{V}$O2

**Figure 5:**
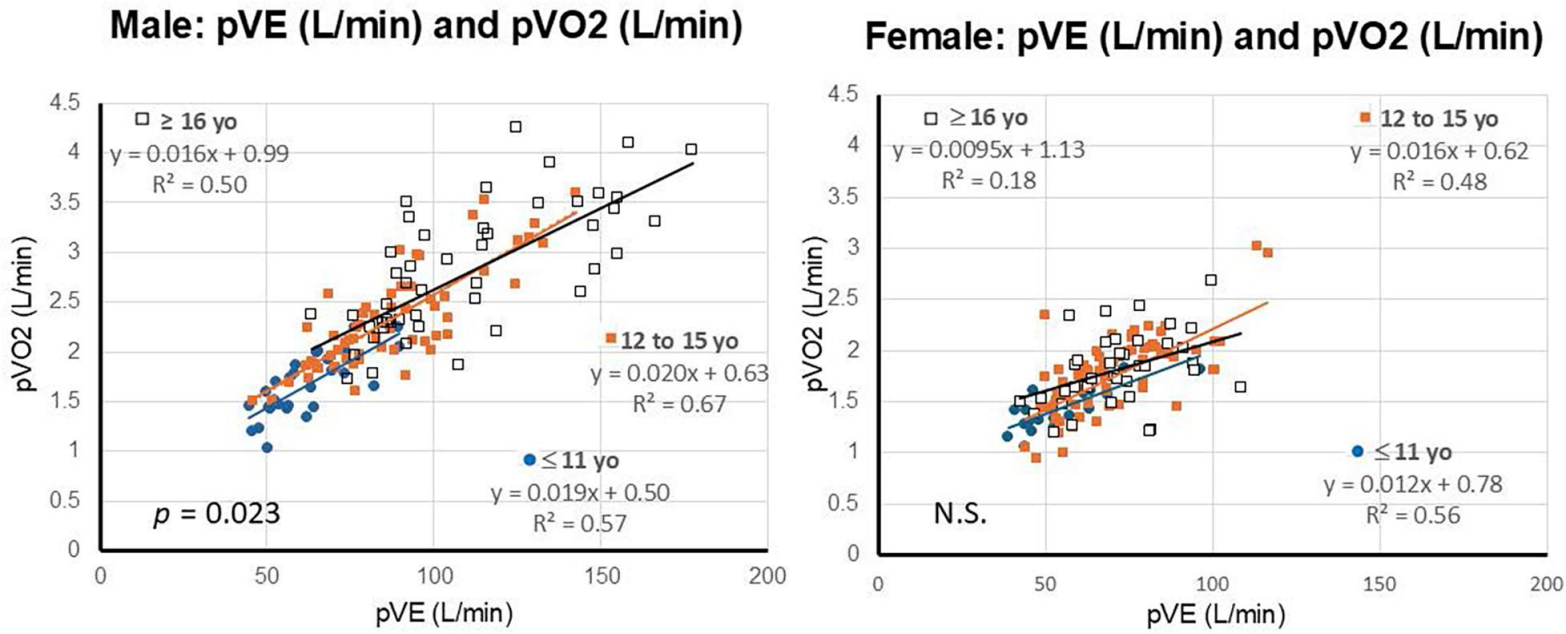
Scattered graphs with regression lines between peak minute ventilation (p$\dot{V}$E) (L/min: *x*-axis) and peak oxygen consumption (p$\dot{V}$O2) (L/mL; *y*-axis) showing ventilatory oxygenation (or central oxygen uptake) efficiency. There is an upward and rightward shift of the dots with increasing ages in males whereas no significant difference is noted in females. Blue closed circle: ≤11 yo, orange closed square: 12–15 yo, and open square: ≥16 yo.

Figure 5 demonstrates very good positive linear correlations between pVE and p$\dot{V}$O2 in both sexes in all age groups except in female ≥16 yo group where the correlation is not as strong (*R*^2^ = 0.18). There is a modest but significant age-dependent upward deviation of the regression line in males, where the regression lines of 12–15 yo and ≥16 yo groups are nearly alike. Age-related upward and rightward shift of scatter plot distribution may reflect continuous growth of lung volume and probably increased central oxygenation efficiency with pubertal growth in males. Female groups show no significant difference in regression lines among different age groups.

#### 3.3.5 Sex differences in age-related changes

**Figure 6:**
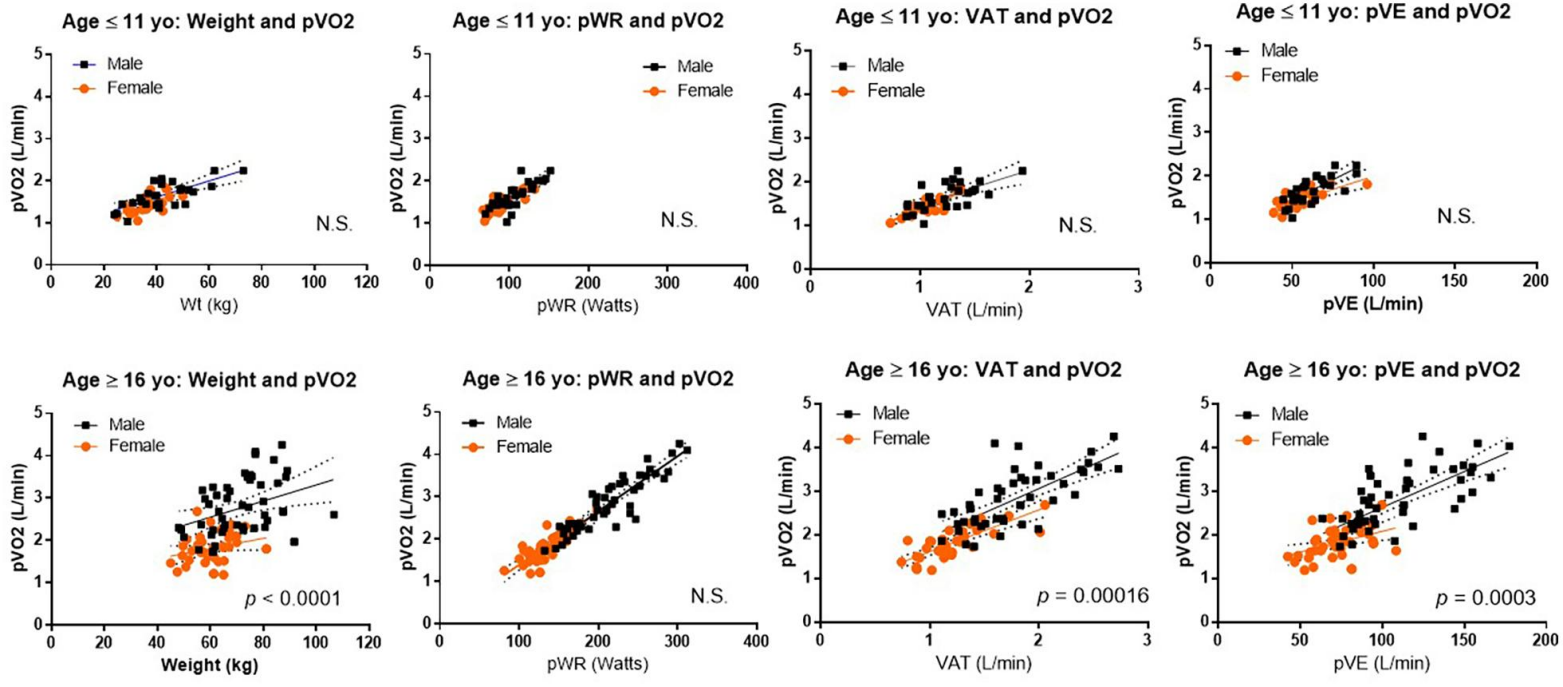
Sex differences of exercise performance at ≤ 11 yo and ≥ 16 yo groups. There is no significant difference between males and females in weight-p$\dot{V}$O2, pWR-p$\dot{V}$O2, VAT-p$\dot{V}$O2, or p$\dot{V}$E- p$\dot{V}$O2 relationships in ≤ 11 yo groups (upper row). Significant sex differences are evident in weight-p$\dot{V}$O2, VAT-p$\dot{V}$O2, and p$\dot{V}$E- p$\dot{V}$O2 relationships in ≥ 16 yo groups (lower row). Regression lines of pWR-p$\dot{V}$O2 are almost identical between males and females regardless of age. Dotted lines indicate 95% confidence intervals.

Age-related sex differences are presented at the youngest (≤11 yo) and the oldest (≥16 yo) age groups (Figure 6). Unlike in age ≤11 yo, significant sex differences are apparent in age ≥16 yo groups in weight-p$\dot{V}$O2, VAT-p$\dot{V}$O2, and p$\dot{V}$E-p$\dot{V}$O2 relationships, where upward and rightward deviation of the regression lines of males compared with those of females, indicating significantly higher oxygen uptake per body weight, higher exercise tolerance beyond AT, and higher oxygen uptake per ventilation, respectively, in older male adolescents. In contrast, regression lines of pWR-p$\dot{V}$O2 of males and females nearly overlap in both age groups except marked upward and left shift of scatter plots of male in age ≥16 yo.

## 4 Discussion

In 303 healthy children and adolescents ranging from 7 to 18 years of age, we studied how age and sex affect exercise performance by comparing three different age groups through peak and submaximal CPET parameters. The two-dimensional analysis provided additional insights into the underlying mechanisms to explain the age and sex-dependent exercise performance. With this combined approach, the changes in exercise performance during adolescence may be characterized by a) age-related somatic growth (increased muscle mass and increased size of internal organs, heart and lungs), b) age-related functional maturation (more efficient oxygen uptake, both central and peripheral, and enhanced tolerance to anaerobic condition in muscles), and c) sex-dependent pubertal changes and subsequent body composition (23).

### 4.1 Enhanced exercise performance with increased somatic growth

Rapid growth of body size or growth spurt commonly occurs during adolescence consisting of increase in bone length and mass, skeletal muscle mass, heart size, intravascular volume, and lung volume, all of which are known to contribute to increased p$\dot{V}$O2 and exercise performance (9, 24). In our cohort, both males and females show physical growth throughout these time periods, but males continue to grow more prominently even after age 15 than females, as shown in Table 1. In females, no significant difference is seen in somatic growth between 12–15 yo and ≥16 yo groups. This growth pattern is in parallel with the changes in peak SBP, absolute peak CPET parameters (p$\dot{V}$O2, pWR, and peak OP), and submaximal parameters (OUES and Δ$\dot{V}$O2/ΔHR), suggesting a positive correlation between exercise performance and physical growth (25, 26). Peak $\dot{V}$E and p$\dot{V}$E/height also increase with physical growth, suggesting growth of lung volume and diffusion surface as well as augmentation of respiratory muscle in both sexes. These findings regarding age-related changes in exercise performance are in agreement with the previously published studies (9, 27).

### 4.2 Age-related functional maturation or improvement in oxygen uptake kinetics

We also identified age-related probable functional maturation in exercise performance, including significantly lower pRER and higher Δ$\dot{V}$O2/ΔWR in younger ages than those in older ages (Table 1). Immaturity of anaerobic metabolism in children compared with older adolescents may be explained by higher proportion of type I (slow twitch) fiber recruitment and reduced anaerobic glycolytic ATP re-phosphorylation in younger individuals (27, 28). Thus, preadolescent children frequently do not reach pRER of 1.1 despite their maximum exercise effort. A recent study by Griffith et al. showed the similar age-related increase of pRER in children from 6 to 18 years of age (29). Higher Δ$\dot{V}$O2/ΔWR in ≤11 yo group than in ≥12 yo groups in both sexes may represent higher oxygen cost or less efficient kinetics of high-energy phosphate metabolism in muscle cells, reflecting immature energy metabolism in younger age groups (30, 31). On the other hand, obese subjects tend to show elevated Δ$\dot{V}$O2/ΔWR because of the excessive weight burden they carry (7). The interpretation of Δ$\dot{V}$O2/ΔWR requires some caution as it is highly dependent on the physiological context; low Δ$\dot{V}$O2/ΔWR also represents impaired aerobic work efficiency or decreased physical conditioning when p$\dot{V}$O2 is low (32).

### 4.3 Sex-dependent effects on exercise performance

In addition to physical growth-dependent increase in CPET parameters, there were considerable sex differences in exercise performance more evident in older groups, especially in ≥16 yo groups (Figure 6). During puberty, androgen generates strong anabolic effects as well as virilization that further increases bone and muscle mass, whereas estrogen enhances deposition of body fat in a special manner (33). Additionally, heart size and stroke volume were noted to be higher in adolescent males (34) as well as lung volume and $\dot{V}$E (35), thus allowing males to exhibit overall better exercise performance than females after puberty. In contrast, female puberty is mainly characterized by increased body fat by estrogen predominance.

Males demonstrate age-dependent augmentation of weight-p$\dot{V}$O2 slope whereas there are no such age-related slope changes in female subgroups (Figure 2). Enhanced skeletal muscle performance in older males is also supported by the age-related changes in regression lines between VAT and p$\dot{V}$O2 (Figure 4), indicating more sustainable muscle metabolism in higher intensity of exercise beyond AT in males (36). This may be explained, in part, by increasing proportion of type II muscle fibers with age in post-pubertal males, which enables muscle to tolerate better under anaerobic conditions (37). This male puberty-induced enhanced skeletal muscle performance at peak exercise may enable older adolescent males to take on a greater pWR. Similar age-related enhanced ventilatory efficiency for oxygenation indicated by the p$\dot{V}$E-p$\dot{V}$O2 relationship is more prominent in males than in females (Figure 5). A larger chest cavity, larger lung volume and higher diffusion capacity, and stronger respiratory muscle in males may explain this phenomenon (38).

The specific body composition in older females is identified as a female puberty-related disadvantage in exercise performance. Despite significant increase of absolute p$\dot{V}$O2 in older females compared with that in ≤11 yo females, weight-indexed p$\dot{V}$O2 (=p$\dot{V}$O2/kg) is significantly lower in older female groups (≥12 yo groups) than in younger females (≤11 yo), suggesting the increased body fat mass in post-pubertal females negatively affecting the weight-indexed value (39). A similar trend was also noted in pOP/kg, VAT/kg, and Δ[$\dot{V}$O2/kg]/ΔHR (Table 1). These female disadvantages may stem largely from fundamental fallacy of using body weight, which we commonly follow in our clinical practice. When p$\dot{V}$O2 is calculated relative to lean body mass instead of total body mass, these sex differences are reduced by approximately one half (40). The difference in peak $\dot{V}$O2 between males and females disappears when pVO2 is indexed by estimated leg muscle mass (41).

### 4.4 Comprehensive interpretation of CPET with peak and submaximal parameters

In addition to interpreting each isolated CPET value, we examined one CPET value in context with another parameter to understand the difference of general exercise trends of the two groups. Figure 2 showed how body weight affects p$\dot{V}$O2, where a distinct age-dependent upward shift of the regression lines was noted only in males. This trend suggests that significant age-dependent functional augmentation of oxygen utilization at a muscle level only in male groups. Figure 3 demonstrated excellent positive correlations between pWR and p$\dot{V}$O2 in all age groups in both sexes where three regression lines were almost identical in all age groups, indicating a relatively consistent trend in ordinary children and adolescents. With this tight relationship, p$\dot{V}$O2 may be reliably estimated by measured pWR in healthy children and adolescents regardless of age, sex, body habitus, stage of puberty, or physical conditioning. Exercise endurance beyond AT was assessed by the relationship between VAT and p$\dot{V}$O2 (Figure 4), which presented significant upward shift of regression lines with age in males, suggesting male pubertal changes providing better endurance under anaerobic conditions (28, 42); this upward shift was relatively modest in females. This difference in oxygen uptake kinetics may largely be attributed to muscle fiber type distribution and its recruitment and substrate utilization although underlying mechanisms are complex (43).

Our combined CPET interpretation with peak and submaximal parameters and two-dimensional analysis may serve as a powerful tool in assessing baseline exercise physiology. Poor exercise capacity is frequently seen in patients with underlying heart disease, which may be due to intrinsic hemodynamic limitation from underlying heart disease and/or physical deconditioning secondary to lack of daily physical activities. It is important to know what contributes to their poor exercise performance as the latter part can be improved by introducing appropriate exercise training (cardiac rehabilitation). However, physiological interactions between involved organs, heart, lung, skeletal muscle, and autonomic nervous system, during exercise are complex, inter-dependent, and yet to be investigated. Further research activities are warranted to delineate these complex mechanisms that comprise exercise performance.

## 5 Limitations

Our study has several important limitations. First, there may be an intrinsic selection bias as our patients came to cardiology clinic for particular reasons, who may not necessarily represent an entirely healthy pediatric population. Second, body compositions, lean body mass and percent body fat, were not directly measured in this study. We only obtained conventional anthropometric measurement and excluded those with BMI > 30 kg/m^2^ to minimize the effects of excessive body fat; the estimation of body composition or fatness only by BMI has an intrinsic limitation. In addition, BMI frequently underestimates actual fatness in younger children; BMI *z*-score is a more accurate measure to represent body fatness in children. As measured weight does not uniformly denote muscle mass, weight indexed-CPET values are inevitably subject to considerable error with individuals with increased fatness. Third, sex maturation and the degree of physical conditioning were not individually assessed in our study. Our age-dependent classification may not accurately reflect the pubertal stages. In fact, wider distribution of weight-p$\dot{V}$O2 relationships in ≥16 yo groups in both sexes (Figure 1) signifies our diverse patient population with variable degree of body habitus and routine exercise involvement. Fourth, muscle oxidative metabolism or oxygen kinetics was not directly measured in this study. Last, a small cohort size limits the power of statistical analysis.

Substantial gaps exist between the science of exercise physiology and clinical CPET in pediatric practice, especially during pubertal ages. We need to acknowledge these existing gaps and should put maximum effort to overcome the discrepancies. Body fatness assessment by bioelectric impedance scale may provide reliable measures of body composition. Despite these limitations, our study characterized the changes in exercise performance during age-related growth and functional maturation by relatively simple methods that can be introduced in routine clinical practice for better understanding of exercise physiology and cardiopulmonary reserve.

## 6 Conclusion

In conventional CPET, we were able to identify three factors affecting age-related augmentation of exercise performance during growth acceleration in adolescence: (1) age-related physical growth, more prominent in males than in females; (2) age-related enhanced functional maturation or improvement in oxygen uptake kinetics and (3) sex-dependent pubertal changes representing enhanced muscle mass, strength, and endurance in older male adolescents (male-dominant pubertal advantage) and increased body fat mass in older female adolescents (female-dominant pubertal disadvantages). A combined analysis of peak and submaximal CPET parameters and two-dimensional analyses utilized in this study enabled mechanistic interpretation of age-related exercise performance in growing children and adolescents, which may be useful when interpreting CPET variables of those with underlying heart disease.

## Data Availability

The datasets generated and/or analyzed during the current study are not publicly available but are available from the corresponding author on reasonable request.
